# Semaglutide and Musculoskeletal Health: A Mendelian Randomization Study Based on GLP-1 Receptor Expression

**DOI:** 10.2174/0118715303422692260617052555

**Published:** 2026-07-02

**Authors:** Dong Li, Xinyu Liang, Zhijun Li, Huafeng Zhang, Hui Li

**Affiliations:** 1 Department of Orthopedics, Tianjin Medical University General Hospital, Tianjin, China

**Keywords:** GLP1R, bone, muscle, obesity, diabetes, musculoskeletal health

## Abstract

**Introduction:**

Semaglutide, a glucagon-like peptide-1 receptor (GLP1R) agonist, is clinically used for glycemic control and weight loss. However, it is still unclear whether it has any impact on bones and muscles. Does semaglutide have any impact on the musculoskeletal system of patients during its use, or does it have any negative effects on them? This study aims to assess the impact of GLP1R on bone and muscle using Mendelian Randomization.

**Methods:**

We employed expression quantitative trait loci (eQTL) data for the GLP1R gene and combined it with genome-wide association studies (GWAS) data for skeletal and muscular traits to conduct Summary-data-based Mendelian Randomization (SMR) analysis. The exposure was defined as the blood expression level of the GLP1R gene, with the eQTL serving as the instrumental variable. Total bone mineral density, osteoporosis, and fractures were used to evaluate bone health, while appendicular lean mass, grip strength, and walking speed assessed muscle quality. Type 2 diabetes and body mass index (BMI) served as positive controls.

**Results:**

The top eQTL rs9283907 was used to predict GLP1R expression. Positive control results confirmed that GLP1R agonists are effective diabetes treatments and can reduce BMI. There were no significant impacts on osteoporosis (beta: 0.00, 95% CI: -0.01, 0.01, *p*=0.416), total bone mineral density (beta: -0.03, 95% CI: -0.19, 0.13, *p*=0.702), fracture (beta: 0.09, 95% CI: -0.10, 0.28, *p*=0.343), walking pace (beta: 0.00, 95% CI: -0.01, 0.06, *p*=0.216), appendicular lean mass (beta: -0.01, 95% CI: -0.08, 0.06, *p*=0.676), or grip strength (beta: -0.02, 95% CI: -0.06, 0.02, *p*=0.425).

**Discussion:**

This Mendelian randomization study provides genetic evidence that GLP-1 receptor expression is not causally associated with altered musculoskeletal health. The findings suggest that semaglutide is unlikely to adversely affect bone metabolism or muscle function regarding the outcomes measured. These results support the musculoskeletal safety profile of semaglutide; however, as genetic associations do not fully capture the clinical pharmacology of the drug, further validation through long-term randomized controlled trials is warranted.

**Conclusion:**

According to our results, high expression of glucagon-like peptide-1 receptor does not lead to bone and muscle loss, which indicates that semaglutide may be safe for the musculoskeletal system.

## 1 INTRODUCTION

Obesity and diabetes are major risk factors for numerous diseases and mortality worldwide. They significantly impact national economies by reducing productivity and life expectancy, increasing disability and healthcare costs [1-3]. The prevalence of these conditions has risen dramatically in recent decades [4]. In the early 2010s, semaglutide, a GLP1R agonist (GLP-1RA), was introduced for treating type 2 diabetes and was found to induce weight loss [5, 6]. Recently, the European Medicines Agency approved a 2.4 mg weekly dose of semaglutide for chronic weight management in individuals with a body mass index (BMI) > 27 kg/m^2^, drawing considerable attention from obese populations [7]. However, side effects such as pneumonia, acute kidney injury, and gastrointestinal reactions have also been reported [7-9]. There have been case reports of rhabdomyolysis following semaglutide use [10]. Patients with hyperglycemia and high BMI already have issues with bone mineral density and muscle mass [11, 12]. Whether semaglutide affects bone and muscle is a concern for researchers and clinicians, and this remains largely unknown [13, 14]. Therefore, we fill this gap by using Mendelian randomization as a research method to investigate whether there is a causal relationship between GLP1 receptors and musculoskeletal function.

Due to the principle of random allocation of genetic variation during meiosis, Mendelian Randomization (MR) is a powerful epidemiological technique that uses genetic variation as an instrumental variable to infer causal relationships between modifiable exposures and disease outcomes, typically avoiding biases that confound observational studies [15]. Summary-data-based Mendelian Randomization (SMR) uses expression quantitative trait loci (eQTL) as an instrumental variable to evaluate the causal relationship between gene expression and outcomes such as diseases. Like traditional MR, the core idea of SMR is that genetic variation is less affected by confounding factors during random allocation, allowing for more accurate causal inferences. Previous analyses of GLP1R suggest that GLP-1RA reduces the risk of diabetic retinopathy and cancer [16, 17]. But there has been no assessment of GLP-1RA on bone and muscle. Therefore, we hope to investigate whether the use of semaglutide has any impact on the musculoskeletal system, or whether there are any negative effects on muscles or bones when using semaglutide to treat blood sugar or for weight loss. In this study, we use GLP1R eQTL data combined with genome-wide association studies (GWAS) data on skeletal and muscular traits to conduct SMR analysis, thus evaluating the impact of semaglutide on bone and muscle.

## 2 MATERIALS AND METHODS

### 2.1 Study Design

In this study, exposure was defined as the expression level of the GLP1R gene in blood, with the instrumental variable predicting exposure being the eQTL. Total bone mineral density, osteoporosis, and fractures were used to evaluate bone health. Appendicular lean mass, grip strength, and walking pace were used to assess muscle quality [18]. Type 2 diabetes and BMI were used as positive controls (Fig. **1**).

### 2.2 eQTL Data

Large-scale genome-wide meta-analysis data for blood eQTL of the GLP1R gene were obtained from the eQTLGen consortium, which included 29,623 European individuals [19]. In order to satisfy the three assumptions of Mendelian randomization (relevance, independence, and exclusion restriction), suitable eQTL instrumental variables were selected based on the following criteria: allele frequency > 1%, genome-wide significance *P* < 5.0×10^-8^, and F-statistic > 10. Independent SNPS with linkage disequilibrium threshold r^2^ < 0.1 were selected as instrumental variables to avoid pleiotropic interference. Additionally, the eQTL had to be within 1 Mb on either side of the gene. The LDlink website (https://ldlink.nih.gov/?tab=ldtrait) was used to detect whether genetic variants were directly associated with confounding factors or outcomes.

### 2.3 Phenotype GWAS

GWAS data for BMI were sourced from the 2018 GIANT Consortium, including 681,275 individuals [20]. Data for type 2 diabetes were from a 2021 study that included 407,609 participants from the UK Biobank [21]. Data on overall Total bone mineral density, involving 56,284 individuals, measured Total bone mineral density (g/cm^2^) using dual-energy X-ray absorptiometry according to standard manufacturer protocols. There was no significant heterogeneity in this meta-analysis (Phet > 0.1) [22]. Fracture data were from a 2019 study that defined fractures as self-reported *via* questionnaires within the past five years, excluding fractures of the skull, face, hands, feet, pathological fractures due to malignancy, and atypical femur fractures [23]. Appendicular lean mass data were from a 2020 study using UK Biobank cohort samples, quantifying appendicular lean mass as the sum of lean mass in the arms and legs [24]. GWAS data for osteoporosis, grip strength, and walking pace were also sourced from the UK Biobank. There was no significant overlap between the samples included in exposure and outcome studies.

### 2.4 SMR and HEIDI Test

SMR analysis was conducted using the SMR software (version 1.3.1) [25]. SMR was used to identify the top eQTLs significantly associated with the gene and to measure the association between gene expression and outcomes. The HEIDI test was performed to detect linkage disequilibrium and horizontal pleiotropy. The significance threshold for the SMR is set at *P* < 0.05/number of tests with Bonferroni correction in this study (P_SMR < 6.25 × 10^-3^). If the P_HEIDI > 0.01, the causal relationship between eQTL and outcome is not confounded by linkage disequilibrium.

We have adhered to the STROBE-MR guidelines and have included the STROBE-MR checklist.

## 3 RESULTS

The top eQTL rs9283907 was used to predict GLP1R expression. Positive control results showed that GLP1R agonists are effective Type 2 diabetes medications (beta: -0.43, 95% CI: -0.74, -0.12, *p*=0.006) and can reduce BMI (beta: -0.10, 95% CI: -0.15, -0.04, *p*=0.003), indicating the validity of the instrumental variable.

Furthermore, GLP1R agonists had no significant impact on osteoporosis (beta: 0.00, 95% CI: -0.01, 0.01, *p*=0.416), total bone mineral density (beta: -0.03, 95% CI: -0.19, 0.13, *p*=0.702), fracture (beta: 0.09, 95% CI: -0.10, 0.28, *p*=0.343), walking pace (beta: 0.00, 95% CI: -0.01, 0.06, *p*=0.216), appendicular lean mass (beta: -0.01, 95% CI: -0.08, 0.06, *p*=0.676), or grip strength (beta: -0.02, 95% CI: -0.06, 0.02, *p*=0.425). There was no significant heterogeneity in the HEIDI test (Fig. **2**). These results suggest that genetically predicted GLP1R expression levels do not significantly affect bones and muscles.

## 4 DISCUSSION

Our results show that high expression of GLP1R can reduce the risk of diabetes and lower BMI without impacting bones and muscles. This suggests that semaglutide is safe for musculoskeletal health.

A recent randomized controlled trial indicated that in adults with increased fracture risk, weekly subcutaneous injections of 1.0 mg semaglutide did not affect changes in P-PINP from baseline to week 52 compared to placebo, suggesting that weekly semaglutide does not increase bone formation [26]. Additionally, a population-based cohort study found that the use of GLP-1RA was not associated with reduced fracture risk compared to users of other antidiabetic drugs [27]. Multiple meta-analyses have also shown that GLP-1RA does not alter fracture risk compared to other antidiabetic medications [28, 29]. These findings are consistent with our study.

In a study of patients with metabolic dysfunction-associated fatty liver disease who received subcutaneous semaglutide for 24 weeks, MRI and functional assessments such as the standing test and walking pace were used to evaluate lumbar muscle volume and body function. The results showed that although lumbar muscle volume decreased, there was no significant change in standing and walking pace in patients treated with low-dose semaglutide. This indicates that despite significant muscle reduction with weight loss, functionality is maintained [30]. Mouse experiments suggest that semaglutide has therapeutic effects on skeletal muscle atrophy, potentially related to reduced ROS accumulation and pro-inflammatory cytokines [31]. Another retrospective study found that 24-week semaglutide treatment improved glycemic control in Japanese patients with type 2 diabetes, reduced body fat content, and did not alter muscle mass [32]. Overall, based on our results and cumulative evidence from randomized controlled trials, policymakers and physicians can be reassured that these drugs are unlikely to increase the risk of osteoporosis and sarcopenia. Our results may play a role in future research on the drug mechanism and clinical application of semaglutide, but the clear function of this drug requires a series of experiments for verification.

Compared to observational studies, this study has clear advantages. By utilizing MR, we effectively used genetic information as a tool for causal inference, making our results statistically persuasive. Clinicians can avoid complex and costly randomized clinical trials, and do not need to worry about common ethical issues in clinical research [33].

## STUDY LIMITATIONS

 However, this study also has limitations. It aims to identify the genetic relationship between GLP1R expression levels and musculoskeletal health, without considering the impact of glucose-lowering and BMI reduction on bones and muscles. In addition, instrumental variables may also be associated with unknown pleiotropy. The study population was mainly European and may not be appropriate for other populations. Gender differences in osteoporosis were not considered. The results need experimental validation and should be interpreted with caution.

## CONCLUSION

The results of our Mendelian randomization study indicate that the high expression of GLP-1 receptors may not have a significant association with musculoskeletal health. This suggests that semaglutide may have no adverse effects on bone metabolism and muscle function. Further research based on the foundation work is needed for a more in-depth exploration.

## Figures and Tables

**Fig. (1):**
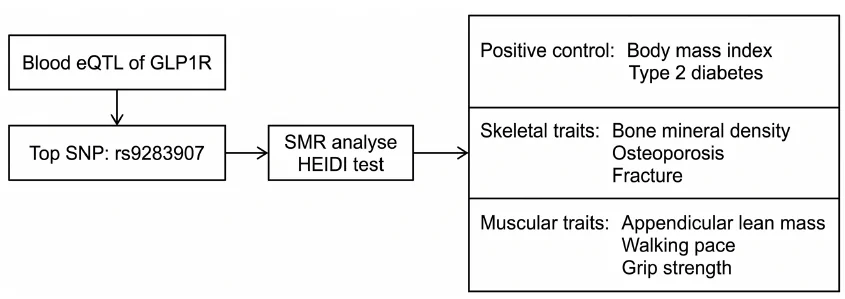
This study analyzes the schematic diagram.

**Fig. (2):**
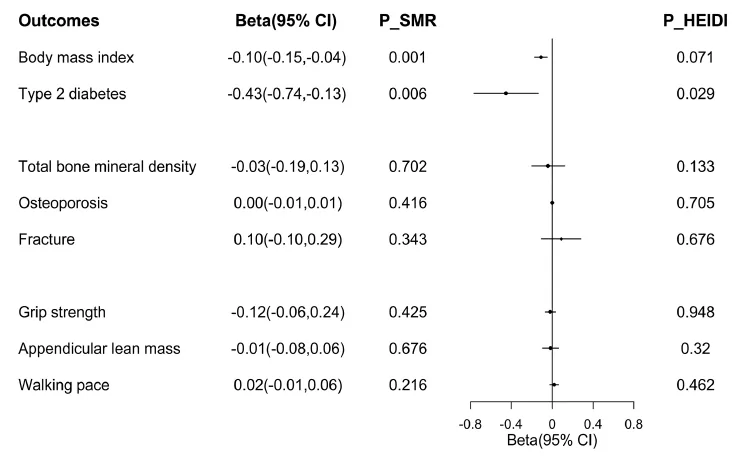
SMR and HEIDI Test.

## Data Availability

Blood eQTL data from the eQTLGen consortium (https://eQTLgen.org/cis-eQTLs.html). GWAS data are available in the IEU database access (https://gwas.mrcieu.ac.uk/). The ID is as follows: ieu-b-40 (BMI), ebi-a-GCST90013892 (Type 2 diabetes), ebi-a-GCST005348 (total bone mineral density), ukb-b-12141 (osteoporosis), ebi-a-GCST006980 (fracture), ukb-b-7478 (grip strength), ebi-a-GCST90000027 (appendicular lean mass), ukb-b-4711 (walking pace).

## LIST OF ABBREVIATION

GLP-1RA: Glucagon-Like Peptide-1 Receptor Agonist
eQTL: Expression Quantitative Trait Loci
SMR: Summary-data-based Mendelian Randomization
BMI: Body Mass Index
MR: Mendelian Randomization

## STANDARDS OF REPORTING

STROBE-MR guidelines and methodology were followed.
